# Oncological outcomes after transanal total mesorectal excision for rectal cancer

**DOI:** 10.1093/bjs/znad168

**Published:** 2023-06-14

**Authors:** Nader K Francis, Marta Penna, Spyridon Dritsas, Harry Kinsey, Brendan Moran, Deborah Nicol, Edward Courtney, Fiona Carter, Sapho Roodbeen, Steve Arnold, Neil Mortensen, Paul White, Roel Hompes, Greg Wynn

**Affiliations:** Department of Colorectal Surgery, Yeovil District Hospital Foundation Trust, Yeovil, UK; Division of Surgery and Interventional Science, UCL, London, UK; Department of Colorectal Surgery, Churchill Hospital, University Hospitals of Oxford, Oxford, UK; Department of Surgery and Cancer, Imperial College London, London, UK; Department of Colorectal Surgery, Yeovil District Hospital Foundation Trust, Yeovil, UK; Department of Colorectal Surgery, Yeovil District Hospital Foundation Trust, Yeovil, UK; Department of Colorectal Surgery, Basingstoke and North Hampshire Hospital, Basingstoke Hospital, Basingstoke, UK; Department of Colorectal Surgery, Worcestershire Royal Hospital, Worcestershire Acute Hospitals NHS Trust, Worcestershire, Worcester, UK; Department of Colorectal Surgery, Royal United Hospital Bath, Bath, UK; Southwest Surgical Training Network Community Interest Company, Yeovil, UK; Department of Colon and Rectal Surgery, Humanitas Clinical and Research Centre, Milan, Italy; Department of Colorectal Surgery, Basingstoke and North Hampshire Hospital, Basingstoke Hospital, Basingstoke, UK; Department of Colorectal Surgery, Churchill Hospital, University Hospitals of Oxford, Oxford, UK; Department of Data Science and Mathematics, University of the West of England, Bristol, UK; Department of Surgery, Amsterdam University Medical Centre, Amsterdam, the Netherlands; ICENI Centre, North Essex Foundation Trust, London, UK

## Introduction

Transanal total mesorectal excision (TaTME) was developed to overcome the technical difficulties of a transabdominal minimal-access approach to resection of low rectal cancer^1–4^. TaTME was reported to be feasible and effective, with the potential advantage of better access to the distal rectum and mesorectum, and theoretical capability of delivering oncologically superior specimens^5,6^.

In the UK, a national training initiative was developed and piloted, confirming the feasibility and safety of this technique within a structured training framework^7,8^. However, a widely publicized moratorium on TaTME^9^ was issued in 2019 in Norway, substantiated by reports of higher-than-expected local recurrence rates, in particular multifocal recurrence. In response to this publication, the Association of Coloproctology of Great Britain and Ireland^10^ issued recommendations in 2020 proposing a pause to the practice of TaTME in the UK.

The aim of the present study was to collate oncological data from English centres that had adopted TaTME to investigate the oncological safety of the technique and factors associated with local recurrence.

## Methods

This was an observational study, using data collated from centres performing TaTME in England. Centres that performed over 10 procedures were identified from the international TaTME registry, and the designated principal investigator at each site was contacted to enter their data into a pro forma developed by the steering committee. Adult patients undergoing TaTME for histologically proven rectal cancer with the intention to cure and no detectable metastases at time of diagnosis were eligible for inclusion.

Oncological data were collected on the following variables. Locoregional recurrence was defined as any recurrent disease in the pelvis in the previous area of dissection, at the anastomotic site, or as pelvic nodal disease^11^. Suspected locoregional recurrence had to be confirmed by imaging (CT/MRI and/or PET). Disease-free survival (DFS) was measured from the date of TaTME for rectal cancer until the date of the first documented pelvic recurrence or development of metastatic disease. Overall survival (OS) was measured from the date of TaTME for rectal cancer until the date of last follow-up or death from any cause.

Demographic, patient, and histopathological data were also collected, as was information on postoperative short-term outcomes including complications (classified according to Clavien and Dindo).

Differences in OS and DFS were compared using Kaplan–Meier curves and analysed using the log rank test. A Cox proportional hazards regression model was used to predict oncological outcomes, adjusted for stratification factors, including tumour site, margin assessment (both distal and circumferential), presence of liver metastases not detected before operation, preoperative administration of radiotherapy, age, sex, and final histological TNM classification (T2, T3, T4 and N1/2 with T0/T1 and N0 respectively as reference).

## Results

A total of 478 TaTMEs were performed in 16 centres in England between February 2013 and September 2021. The median age of the patients was 66 (i.q.r. 59–73) years and 372 patients (77.8 per cent) were men. Most patients had an ASA grade of II (18.2 per cent) or III (61.9 per cent). Median BMI was 28 (i.q.r. 22–31) kg/m^2^, and the median tumour height from the anal verge on MRI was 7 (6–9) cm. Some 153 patients (33.0 per cent) received neoadjuvant therapy; 75 per cent had long-course chemoradiotherapy and 24 per cent short-course radiotherapy (*Table 1*). Restorative anterior resection was undertaken in 426 patients (89.1 per cent), and 46 (9.6 per cent) had abdominoperineal excision (44 intersphincteric, 9.2 per cent). The vast majority of procedures (85.9 per cent) involved the use of an Airseal device (ConMed Surgiquest AirSeal^®^ [Swindon, UK] iFS—Insufflator). Purse-string failure was reported in 15 patients (3.1 per cent).

**Table 1:** Demographics and short-term outcomes of patients undergoing transanal total mesorectal excision in England (2013–2021)

	No. of patients (*n* = 478)
**Tumour location**	
Anterior	189 (44.3)
Lateral	82 (19.2)
Posterior	145 (34.0)
Annular	11 (2.6)
Missing	51
**Neoadjuvant therapy**	
No	325 (68.0)
Yes	153 (32.0)
**Operation type**	
Anterior resection	112 (23.5)
Low anterior resection	314 (65.8)
Intersphincteric abdominoperineal excision	44 (9.2)
Other	7 (1.5)
Missing	1
**Insufflation type**	
Standard	31 (6.8)
Airseal^®^	411 (90.1)
Path reservoir	6 (1.3)
Endoflator	8 (1.8)
Missing	22
**Tumour category**	
T0	34 (7.1)
T1	45 (9.4)
T2	164 (34.3)
T3	228 (47.7)
T4	7 (1.5)
**Node category**	
N0	314 (65.7)
N1	111 (23.2)
N2	53 (11.1)
**Specimen quality**	
Intact	397 (92.8)
Minor defects	22 (5.1)
Major defects	9 (2.1)
Missing	50
**Resection margin**	
R0	460 (96.2)
R1	18 (3.8)
**Purse-string failure**	
No	460 (96.8)
Yes	15 (3.2)
Missing	3
**Anastomotic leak**	
No	371 (87.1)
Yes	55 (12.9)
Missing	31
**Clavien–Dindo complication grade**	
0	308 (64.4)
I	46 (9.6)
II	56 (11.7)
IIIa	14 (2.9)
IIIb	45 (9.4)
IV	6 (1.3)
V	3 (0.6)
**Unplanned return to theatre**	
No	419 (87.7)
Yes	59 (12.3)
**30-day readmission**	
No	428 (89.5)
Yes	50 (10.5)

Values are *n* (%). Airseal^®^ (Swindon, UK).

### Short-term clinical outcomes

At 30 days, 308 patients (64.4 per cent) had no recorded complications. The 30-day mortality rate was 0.6 per cent (3 deaths). Overall, 55 of 426 patients with an anastomosis (12.9 per cent) had an anastomotic leak. Thirty patients (10.5 per cent) were readmitted to hospital within 30 days, mostly owing to anastomotic leak (8, 16 per cent), high stoma output with acute kidney injury (8, 16 per cent), and intra-abdominal or pelvic collection/abscess (5, 10 per cent).

At pathological staging, 392 of 478 patients (82.0 per cent) had T2–T3 cancers and 164 (34.3 per cent) had N1–N2 disease. The vast majority of TME specimens (397, 92.8 per cent) were intact (grades I and II). The median number of lymph nodes harvested was 19 (13–26). Three patients (0.6 per cent) had a positive distal margin and 15 (3.1 per cent) a positive circumferential resection margin. Median distal and circumferential resection margins were 16 (i.q.r. 10–30) and 10 (5–15) mm respectively; 460 patients (96.2 per cent) had R0 resections.

Median follow-up from the date of surgery was 36 months. At 24 months, DFS and OS rates were 95.9 and 92.7 per cent respectively. The overall mortality rate was 16.3 per cent (80 patients), with a mean(s.d.) interval between surgery and death of 31(20) months.

Local recurrence was diagnosed in 35 patients (7.3 per cent), with the majority of recurrences (25, 72.2 per cent) isolated to the pelvis; 10 (28 per cent) were at the bowel wall/anastomosis or presacral region. The mean(s.d.) interval between surgery and local recurrence diagnosis was 25.8 ± 16.5 months with 1 (2.8 per cent) recurring within 6 months; one patient (2.8 per cent) developed recurrence within 6 months and nine after 36 months. Overall, 81 patients (16.5 per cent) developed metastatic disease, mostly in the liver (61.7 per cent) and lung (48.1 per cent).

**Fig. 1:**
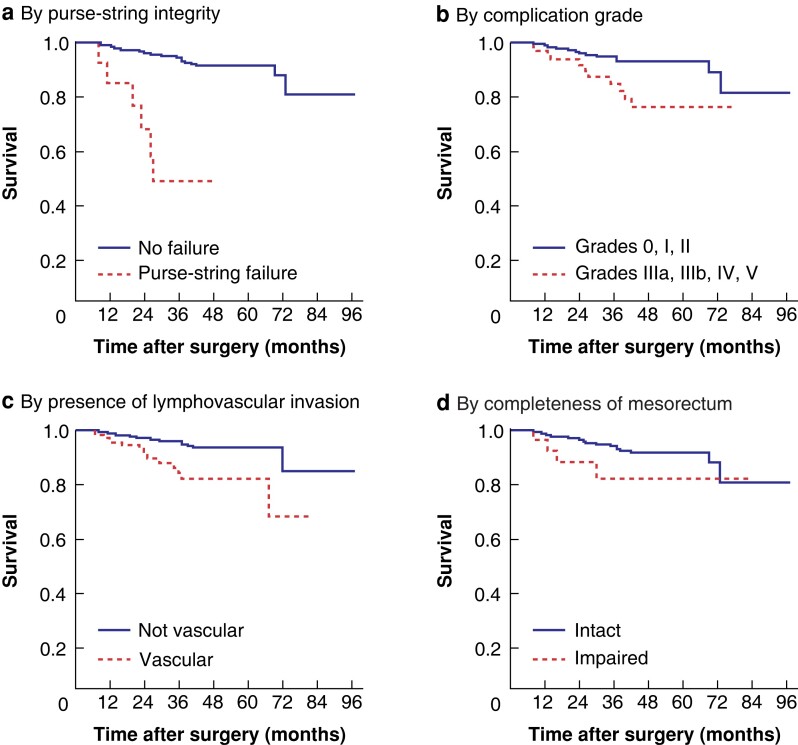
Kaplan–Meier curves showing factors predicting disease-free survival after transanal total mesorectal incision **a** Purse-string failure (*P* < 0.001), **b** Complications above Clavien–Dindo grade III (*P* < 0.001), **c** lymphovascular invasion (*P* < 0.001), and **d** incomplete mesorectum at pathological assessment (*P* = 0.003) (log rank test). Purse string failure 15 (3.2%); complications >III 65 (13%); lymphovascular invasion 119 (24.9%); CRM 18 (3.8%).

Multivariable Cox proportional hazards analysis for DFS obtained from purposeful variable selection found that purse-string failure (HR 14.01; *P* < 0.001), complications with a Clavien–Dindo grade of IIIa, IIIb or IV (HR 4.97; *P* < 0.001), lymphovascular invasion (HR 4.07; *P* < 0.001), and an incomplete mesorectum at pathological assessment (HR 5.48; *P* = 0.003) were independent predictors of local recurrence (*Fig. 1*).

## Discussion

This study demonstrated an OS rate of 92.7 per cent at 1 year after TaTME performed in 16 English centres, with local recurrence and DFS rates of 7.3 and 95.9 per cent respectively.

Purse-string failure was identified as a significant risk factor for local recurrence and DFS. This was reported in only 15 patients (3 per cent) in the present cohort, but is a recognized technical failure in TaTME. In a national TaTME training programme involving structured mentoring of procedures, purse-string failure was observed in 4 of 25 operations at the early stage of training, all of which were corrected by the trainers^8^. This reinforces the importance of mentorship during the early phase of the learning curve, and emphasizes the importance of a reliable purse-string suture to avoid leakage of faecal material and/or cancer cells during the perineal dissection.

In the published literature on TaTME, the locoregional recurrence rate varies from 2 to 16 per cent, including of 11.6 per cent in the Norwegian study^9^. A higher locoregional recurrence rate has been reported during the early phases of implementation of TaTME, again highlighting the importance of structured training and prolonged proctorship^12^. Our study describes the initial development and implementation of this complex technique, and as such all centres submitted data from their initial experience. Only five centres received structured mentorship for five procedures during the national pilot study in England^7^. Although formal training programmes would hypothetically have been ideal for all surgeons learning TaTME, the present results highlight the wider challenges that remain in developing technical expertise when introducing novel complex techniques^13^.

The findings of this multicentre study support the oncological safety of a transanal approach to low rectal cancer. However, careful selection of patients and experienced trained surgeons are required to avoid adverse events.

## Data Availability

The authors confirm that the data supporting the findings of this study are available in the article.
